# Hyperbaric oxygen therapy applied research in traumatic brain injury: from mechanisms to clinical investigation

**DOI:** 10.1186/2045-9912-4-18

**Published:** 2014-12-04

**Authors:** Yang Wang, Dongdong Chen, Gang Chen

**Affiliations:** Department of Neurosurgery, The First Affiliated Hospital of Suzhou University, Suzhou, People’s Republic of China

**Keywords:** Hyperbaric oxygen therapy, Traumatic brain injury, Animal experiments, Clinical trials, Oxygen toxicity

## Abstract

Traumatic brain injury (TBI) is the leading cause of mortality and morbidity for millions of young people and military personnel around the world every year. Regardless of severity, neurological dysfunction is a sequela of TBI. Although many preclinical and clinical trials have been carried out to explore its underlying pathophysiology, few effective treatment options have been used to ameliorate the prognosis of TBI, particularly with regard to the recovery of neurological deficits. Translational medicine has increasingly emphasized secondary brain injury, as distinguished from the mechanical damage occurring at the moment of traumatic impact; this includes cerebral ischemia, vasospasm, metabolic dysfunction, oxygenation absence and edema. Hyperbaric oxygen therapy (HBOT) is defined as the inhalation of pure oxygen in a hyperbaric chamber that is pressurized to greater than 1 atm. High concentrations of oxygen in the blood could affect brain tissue hypoxia readily thereby avoiding neuronal cell death through increased cerebral oxygen metabolism. Therefore, HBOT has been suggested as a scientific and effective treatment for TBI. The effectiveness and feasibility of HBOT has been confirmed by several studies. Following the widespread application of HBOT in cerebrovascular diseases and TBI, non-standard therapies frequently occur in primary care institutions, causing great controversy. The systematic analysis of the progress of both animal and clinical studies in this article provides the basis for further study of HBOT.

## Review

Trauma is the major cause of mortality and morbidity in young people and military personnel (blast-induced trauma), with more than 50% of deaths having been attributed to traumatic brain injury (TBI). The morbidity caused by TBI carries a tremendous burden for both families and society**.** Surgical treatment, such as hematoma or contusion focus removal, is used for saving lives, but cannot improve the prognosis [1, 2]. The general consensus is that efficient treatments should focus on secondary brain injury. Previously, therapies concentrated on the stabilization of blood and intracranial pressure (ICP). They generally involved the administration of neuroprotective drugs as well as rehabilitation training, though they neglected the hypoxic state of brain tissue after TBI. Several studies have shown that secondary ischemic injury exists in brain tissue in the early stages of TBI and that it is an important contributor to morbidity and mortality. Therefore, an oxygen-directed therapy guided by specific monitoring devices may contribute to reducing mortality and thus improve the outcome for TBI patients [3]. Previous studies have confirmed the theoretical viability of hyperbaric oxygen therapy (HBOT) and investigated the potential mechanism of action and effect of HBOT. Cells in the central nervous system (CNS) rely exclusively on aerobic metabolism and need a high supply of oxygen. The high metabolic rate is mainly associated with neuronal signal transduction, such as synaptic transmission, action potential and nerve excitability. With cerebral hypoxia, neuronal cell death becomes inevitable [4]. Current and promising studies have shown that hypoxia is another therapeutic target in TBI patients. Although some controversy exists on the role of hyperoxia in major brain injury, in our experience, severe complications are rarely observed and are generally reversible. Normobaric oxygen (NBO) and hyperbaric oxygen (HBO) are the two main accepted therapies and HBOT has a significantly more robust effect. Zhou *et al*. have shown that HBOT reduces the ischemic loss of neurons in the hippocampus and have suggested that it improves the neurobehavioral outcome [5, 6]. In our review, we discuss the key role of HBOT in treating TBI on the basis of animal studies, clinical research, complications from HBOT and oxygen toxicity.

## Mechanisms of hyperbaric oxygen therapy in traumatic brain injury

Although HBOT is widely used in medicine and is very effective in nervous system diseases, its underlying mechanism is poorly understood. Hyperbaric oxygen therapy involves breathing pure oxygen in a pressure (i.e. greater than 1 atm) chamber, and is used to increase the amount of oxygen in the blood. Typical HBOT regimens use a pressure of 1.5 or 2.5 atm for consecutive periods lasting 30–90 minutes, repeated multiple times. The consecutive periods and number of repeated sessions vary widely, but it is important to select the appropriate oxygen concentration, oxygen inhalation mode, inhalation time and treatment times based on the specific circumstance [7]. Hyperbaric oxygen therapy is used in many hyperbaric centers to treat severe TBI. In addition, the use of HBOT is widespread in primary care hospitals in China. However, the usefulness of HBOT as a definite post-operative therapeutic regimen, among the conservative or adjuvant treatments that are available, has been proved beyond doubt.

Hyperbaric oxygen therapy likely treats TBI via several different pathophysiological mechanisms [8, 9]: (1) HBOT increases arterial oxygen pressure and brain tissue oxygen levels; (2) some studies have suggested that the diffusion rate and effective diffusion distance of oxygen are also increased; (3) vasoconstriction capacity leads to low cerebral blood flow, which is accompanied by improved consciousness as the result of edema and ICP reduction; (4) not only does HBOT accelerate collateral circulation to protect neurons from ischemic death, but it also repairs the damaged microvessels, thereby simultaneously stimulating angiogenesis and neurogenesis; (5) HBOT can prevent a large microthrombus from forming, while also simultaneously promoting their absorption. The underlying potential of neurological function recovery in TBI patients decreases if the intervention with HBOT is delayed. Therefore, it is essential that HBOT is used in patients as soon as possible.

## Animal studies

**Figure 1:**
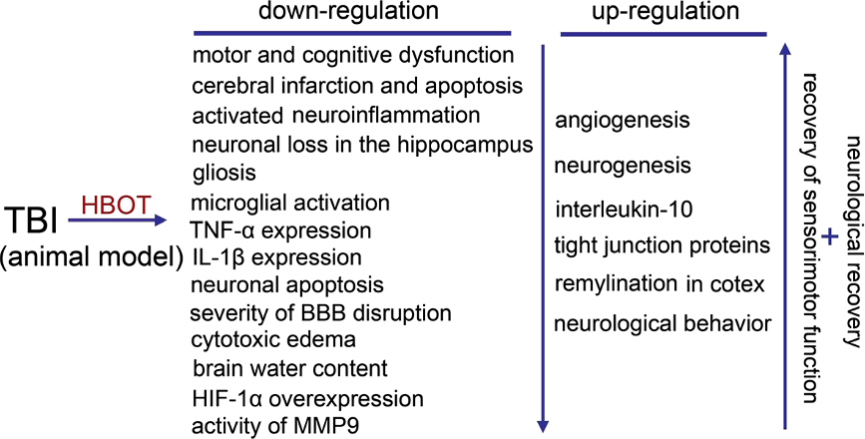
**The possible mechanisms of hyperbaric oxygen (HBO) exerting neuroprotection in traumatic brain injury (TBI).**

Animal studies generally belong to the category of preclinical studies. Comparing experimental results can be relatively difficult on account of the differences in experimental conditions, experimental design and the methods used in different research centers. Therefore, in the future we look forward to conducting novel animal experiments to verify the neuroprotection offered by HBO in TBI using a unified standard. In this article we have reviewed, summarized and analyzed several valuable animal studies related to HBOT as used in animal models (Figure 1).

Both classical and cutting-edge theories are included in our review: (1) HBOT exerts a neuroprotective effect and improves prognosis following blast-induced TBI by promoting the metabolism of local neurons, inhibiting brain edema, protecting the integrity of the blood–brain barrier (BBB), decreasing cell apoptosis, and inhibiting the accumulation of inflammatory cells. Furthermore, timely intervention, i.e. within 1 week of injury, may be more conducive to improving the prognosis of patients with blast-induced TBI [10]; (2) dynamic contrast-enhanced magnetic resonance imaging, diffusion-weighted imaging, and venous clinical severity scores in experimental TBI have suggested that HBOT could improve the impaired BBB and cytotoxic edema following TBI and promote the recovery of neurons [11]; (3) early and timely HBOT intervention can have a more robust effect than delayed intervention, as hypoxia-inducible factor 1-alpha is inhibited and the percentage of apoptotic cells in brain tissue declines dramatically [12]; (4) trauma-associated neurological impairment regressed significantly following 3 weeks of repeated HBOT, a process that is mediated by pronounced remyelination in the ipsilateral injured cortex, as substantiated by the associated recovery of sensorimotor function. Furthermore, this assumption is confirmed by a pronounced increase in myelin basic protein isoforms [13]; (5) a single intervention with HBO has a time limitation of 12 h post-TBI, while the superimposition of multiple HBO treatments can extend the post-TBI delivery time window [14]; (6) in the acute stage, HBOT may improve the outcome of TBI in rats by inhibiting activated inflammation and gliosis, while both angiogenesis and neurogenesis are stimulated [15]; (7) in mice, interleukin-10 plays an important role in the neuroprotective effect of HBOT against TBI, which is associated with resisting neuroinflammation [16]. Above all, the neuroprotective effect of HBOT against TBI has been observed by comparing superficial phenomena and from the levels of some specific proteins, signaling pathways and targeting genes. It is important that the experimental design of prospective animal studies should reach the standards and requirements of clinical trials.

## Clinical trials

Any drug or therapeutic technique needs abundant clinical trials to be validated. So far, HBOT against TBI has undergone Phase II clinical trials. Hypoxic episodes are common events after severe TBI, and most are independent of ICP alterations. In addition, most hypoxic episodes occur while cerebral perfusion and mean arterial pressure are within the accepted range. When cerebral perfusion pressure is < 60 mmHg, the frequency of hypoxic episodes increases significantly [17]. Furthermore, an increased frequency of hypoxic episodes is associated with a poor functional outcome.

Recently, monitoring the partial pressure of oxygen in brain tissue (PbtO_2_) has gained in prominence and the up-regulation of PbtO_2_ is direct evidence of HBOT effectiveness.

A series of factors, including HBOT intervention, PbtO_2_ up-regulation, and improvement of functional outcome, form the foundation of HBOT in TBI patients. In TBI patients, oxygen metabolism can be evaluated using brain tissue oxygen probes. Interestingly, the duration and frequency of cerebral hypoxia episodes decrease in brain tissue according to oxygen-guided therapy based on a standard algorithm [18]. In conclusion, brain tissue oxygenation is associated with clinical outcome and physiological parameters in TBI patients. Hence, we make great efforts to exceed the threshold value of PbtO_2_.

**Figure 2:**
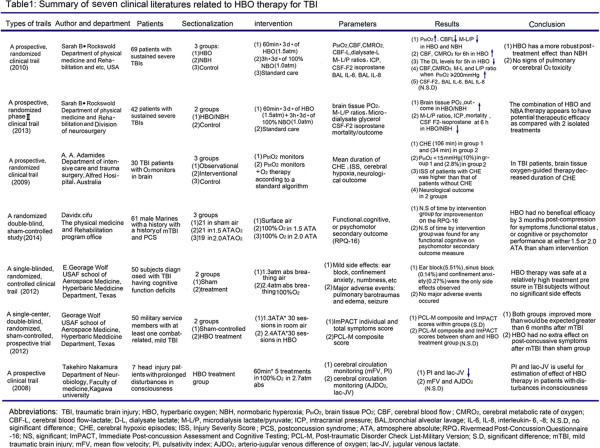
**Summary of seven clinical literatures related to HBO therapy for TBI.**

We systematically analyzed the latest clinical trials concerned with the use of HBOT in TBI patients and the relevant conclusions are shown in Figure 2: (1) when comparing physiological parameters and neurological function scores between control and treatment groups, HBOT is effective in severe TBI and improves patients’ prognosis; (2) in general, HBOT exerts a powerful effect on NBO under resting conditions by ensuring that PbtO_2_ exceeds the threshold value. Besides, the combination of HBO and NBO appears to have a more robust effect than a single HBO or NBO treatment [6]; (3) as HBOT is used widely, the side effects of repeated HBO treatment should be taken into account. Fortunately, when HBOT interventions under specific conditions are applied properly, only a few major adverse events have been observed, such as pulmonary barotrauma, pulmonary edema and seizures. Even though some mild side effects and complications have occurred, such as ear blockage, headache or chest pain, these self-limiting symptoms are seemingly reversible. Thus, HBOT can be considered safe when treating severe TBI [18–24].

Unfortunately, agreement that HBOT has a positive effect on TBI has not yet been reached due to the difference in external conditions: (1) the lack of conformity of patients’ clinical data in the literature may affect our judgment of the therapeutic value of HBOT; (2) the non-standard intervention methods used in primary healthcare increase the risk of complications; (3) obtaining a large sample is extremely costly, especially for smaller research centers. The fewer the cases included in a trial, the lower the credibility of the conclusions drawn from it. Although clinical trials face many ethical and funding challenges, what is to be gained from them justifies their continued use.

## Oxygen toxicity

Oxygen is a cornerstone of modern clinical practice and one of the most frequently used therapeutic agents. Oxygen toxicity is one of the problems encountered in clinical work. Extended exposure to high concentrations of oxygen at greater than 1 atm results in CNS and pulmonary toxicity. Seizures are the most dramatic and dangerous manifestation of oxygen toxicity in the CNS, but they can be reversed without causing significantly serious neurological damage if oxygen inhalation is reduced. Two major adverse events – pulmonary barotrauma and edema – have been observed in oxygen therapy in previous studies, though these serious complications occurred under specific circumstances and in specific patient cohorts [22, 25]. Severe complications or mild side effects in TBI patients have not been reported.

## Conclusions

Increasing evidence has shown that HBOT is a key contributor in the treatment of TBI and occupies an important place in modern neurosurgery. We believe that HBOT is going to be increasingly accepted by patients and approved by clinicians. Apart from TBI, other cerebrovascular diseases, such as intracerebral hemorrhage and high-grade subarachnoid hemorrhage, also have significant therapeutical indications to improve the prognosis, although multiple-system organ failure due to irregular interventions gives rise to concerns. Although HBOT has entered a mature phase, whether in preclinical or clinical studies, further research should be undertaken into the mechanisms and efficacy of HBOT, as it could offer a clinically promising therapeutic approach to TBI.

## Abbreviations

TBI: Traumatic brain injury
HBOT: Hyperbaric oxygen therapy
ICP: Intracranial pressure
CNS: Central nervous system
NBO: Normobaric oxygen
HBO: Hyperbaric oxygen
BBB: Blood–brain barrier
PbtO_2_: Partial pressure of oxygen in brain tissue.

## References

[1] Chua KS, Ng YS, Yap SG, Bok CW (2007). A brief review of traumatic brain injury rehabilitation. Ann Acad Med Singapore.

[2] Flanagan SR, Cantor JB, Ashman TA (2008). Traumatic brain injury: future assessment tools and treatment prospects. Neuropsychiatr Dis Treat.

[3] Beynon C, Kiening KL, Orakcioglu B, Unterberg AW, Sakowitz OW (2012). Brain tissue oxygen monitoring and hyperoxic treatment in patients with traumatic brain injury. J Neurotrauma.

[4] Hyder F, Patel AB, Gjedde A, Rothman DL, Behar KL, Shulman RG (2006). Neuronal-glial glucose oxidation and glutamatergic-GABAergic function. J Cereb Blood Flow Metab.

[5] Zhou Z, Daugherty WP, Sun D, Levasseur JE, Altememi N, Hamm RJ, Rockswold GL, Bullock MR (2007). Protection of mitochondrial function and improvement in cognitive recovery in rats treated with hyperbaric oxygen following lateral fluid-percussion injury. J Neurosurg.

[6] Rockswold SB, Rockswold GL, Zaun DA, Zhang X, Cerra CE, Bergman TA, Liu J (2010). A prospective, randomized clinical trial to compare the effect of hyperbaric to normobaric hyperoxia on cerebral metabolism, intracranial pressure, and oxygen toxicity in severe traumatic brain injury. J Neurosurg.

[7] McDonagh M, Helfand M, Carson S, Russman BS (2004). Hyperbaric oxygen therapy for traumatic brain injury: a systematic review of the evidence. Arch Phys Med Rehabil.

[8] Sánchez EC (2013). Mechanisms of action of hyperbaric oxygenation in stroke: a review. Crit Care Nurs Q.

[9] Gill AL, Bell CN (2004). Hyperbaric oxygen: its uses, mechanisms of action and outcomes. QJM.

[10] Zhang Y, Yang Y, Tang H, Sun W, Xiong X, Smerin D, Liu J (2014). Hyperbaric oxygen therapy ameliorates local brain metabolism, brain edema and inflammatory response in a blast-induced traumatic brain injury model in rabbits. Neurochem Res.

[11] Wei XE, Li YH, Zhao H, Li MH, Fu M, Li WB (2014). Quantitative evaluation of hyperbaric oxygen efficacy in experimental traumatic brain injury: an MRI study. Neurol Sci.

[12] Yang Y, Zhang YG, Lin GA, Xie HQ, Pan HT, Huang BQ, Liu JD, Liu H, Zhang N, Li L, Chen JH (2014). The effects of different hyperbaric oxygen manipulations in rats after traumatic brain injury. Neurosci Lett.

[13] Kraitsy K, Uecal M, Grossauer S, Bruckmann L, Pfleger F, Ropele S, Fazekas F, Gruenbacher G, Patz S, Absenger M, Porubsky C, Smolle-Juettner F, Tezer I, Molcanyi M, Fasching U, Schaefer U (2014). Repetitive long-term hyperbaric oxygen treatment (HBOT) administered after experimental traumatic brain injury in rats induces significant remyelination and a recovery of sensorimotor function. PLoS One.

[14] Wang GH, Zhang XG, Jiang ZL, Li X, Peng LL, Li YC, Wang Y (2010). Neuroprotective effects of hyperbaric oxygen treatment on traumatic brain injury in the rat. J Neurotrauma.

[15] Lin KC, Niu KC, Tsai KJ, Kuo JR, Wang LC, Chio CC, Chang CP (2012). Attenuating inflammation but stimulating both angiogenesis and neurogenesis using hyperbaric oxygen in rats with traumatic brain injury. J Trauma Acute Care Surg.

[16] Chen X, Duan XS, Xu LJ, Zhao JJ, She ZF, Chen WW, Zheng ZJ, Jiang GD (2014). Interleukin-10 mediates the neuroprotection of hyperbaric oxygen therapy against traumatic brain injury in mice. Neuroscience.

[17] Chang JJ, Youn TS, Benson D, Mattick H, Andrade N, Harper CR, Moore CB, Madden CJ, Diaz-Arrastia RR (2009). Physiologic and functional outcome correlates of brain tissue hypoxia in traumatic brain injury. Crit Care Med.

[18] Adamides AA, Cooper DJ, Rosenfeldt FL, Bailey MJ, Pratt N, Tippett N, Vallance S, Rosenfeld JV (2009). Focal cerebral oxygenation and neurological outcome with or without brain tissue oxygen-guided therapy in patients with traumatic brain injury. Acta Neurochir (Wien).

[19] Rockswold SB, Rockswold GL, Zaun DA, Liu J (2013). A prospective, randomized Phase II clinical trial to evaluate the effect of combined hyperbaric and normobaric hyperoxia on cerebral metabolism, intracranial pressure, oxygen toxicity, and clinical outcome in severe traumatic brain injury. J Neurosurg.

[20] Wortzel HS, Arciniegas DB, Anderson CA, Vanderploeg RD, Brenner LA (2012). A phase I study of low-pressure hyperbaric oxygen therapy for blast-induced post-concussion syndrome and post-traumatic stress disorder: a neuropsychiatric perspective. J Neurotrauma.

[21] Cifu DX, Walker WC, West SL, Hart BB, Franke LM, Sima A, Graham CW, Carne W (2014). Hyperbaric oxygen for blast-related postconcussion syndrome: three-month outcomes. Ann Neurol.

[22] Wolf EG, Prye J, Michaelson R, Brower G, Profenna L, Boneta O (2012). Hyperbaric side effects in a traumatic brain injury randomized clinical trial. Undersea Hyperb Med.

[23] Wolf G, Cifu D, Baugh L, Carne W, Profenna L (2012). The effect of hyperbaric oxygen on symptoms after mild traumatic brain injury. J Neurotrauma.

[24] Nakamura T, Kuroda Y, Yamashita S, Kawakita K, Kawai N, Tamiya T, Itano T, Nagao S (2008). Hyperbaric oxygen therapy for consciousness disturbance following head injury in subacute phase. Acta Neurochir Suppl.

[25] Domachevsky L, Pick CG, Arieli Y, Krinsky N, Abramovich A, Eynan M (2012). Do hyperbaric oxygen-induced seizures cause brain damage?. Epilepsy Res.

